# Ebola Virus RNA in Semen from an HIV-Positive Survivor of Ebola

**DOI:** 10.3201/eid2304.161743

**Published:** 2017-04

**Authors:** Lawrence J. Purpura, Emerson Rogers, April Baller, Stephen White, Moses Soka, Mary J. Choi, Nuha Mahmoud, Christine Wasunna, Moses Massaquoi, Jomah Kollie, Straker Dweh, Philip Bemah, Victor Ladele, Jonathan Kpaka, Mary Jawara, Margaret Mugisha, Onyekachi Subah, Mylene Faikai, Jeff A. Bailey, Pierre Rollin, Barbara Marston, Tolbert Nyenswah, Alex Gasasira, Barbara Knust, Stuart Nichol, Desmond Williams

**Affiliations:** Centers for Disease Control and Prevention, Atlanta, Georgia, USA (L.J. Purpura, M.J. Choi, P. Rollin, B. Marston, B. Knust, S. Nichol, D. Williams);; Ministry of Health, Monrovia, Liberia (E. Rogers, M. Soka, M. Massaquoi, P. Bemah, T. Nyenswah);; World Health Organization, Monrovia (A. Baller, N. Mahmoud, J. Kollie, V. Ladele, M. Mugisha, A. Gasasira);; Academic Consortium Combating Ebola in Liberia, Monrovia (S. White, C. Wasunna, S. Dweh, J. Kapka, O. Subah, J.A. Bailey);; Men’s Health Screening Program, Monrovia (M. Jawara, M. Faikai)

**Keywords:** Ebola virus disease, Ebola virus, viruses, virus RNA, semen, HIV, HIV/AIDS and other retroviruses, co-infection, zoonoses, Ebola treatment unit, Liberia

## Abstract

Ebola virus is known to persist in semen of male survivors of Ebola virus disease (EVD). However, maximum duration of, or risk factors for, virus persistence are unknown. We report an EVD survivor with preexisting HIV infection, whose semen was positive for Ebola virus RNA 565 days after recovery from EVD.

In March 2015 in Liberia, unprotected sexual intercourse was strongly suspected in the transmission of Ebola virus disease (EVD) from a male survivor of EVD to his female partner (*1*). Results of Ebola RNA sequence analysis for a semen sample from the survivor 199 days after onset of illness and blood samples from the female patient were consistent with direct transmission.

In July 2015, the Liberian Ministry of Health established the Men’s Health Screening Program to offer semen testing for Ebola virus and behavioral counseling on safe sexual practices to male survivors of EVD to the Ebola response (*2*). We report a survivor of EVD who had a preexisting HIV infection whose semen was positive for Ebola virus RNA 565 days after recovering from this disease.

On August 27, 2014, a 48-year-old man with a history of HIV infection who was receiving antiretroviral therapy was admitted to an Ebola treatment unit (ETU) in Monrovia, Liberia, with a 1-week history of fever, chills, and weakness and a 2-day history of vomiting and diarrhea. The next day, he had a positive result for Ebola virus in blood by real-time reverse transcription PCR (RT-PCR), with a cycle threshold (C_t_) of 32.39. While in the ETU, he continued his antiretroviral therapy. The patient was discharged on September 8, 2014, after showing a negative result for Ebola virus in blood by RT-PCR.

When the patient was first given a diagnosis of infection with HIV-1 in October 2009 (CD4 cell count 46/μL) (Figure), he was given ART with zidovudine/lamivudine/nevirapine and trimethoprim/sulfamethoxazole for prophylaxis against opportunistic infections. On March 25, 2010, he was given a diagnosis of co-infection with HIV-1 and HIV-2, and his ART regimen was changed to zidovudine/lamivudine/lopinavir plus ritonavir because HIV-2 strains are typically resistant to non-nucleoside reverse transcription inhibitors, such as nevirapine. His CD4 cell count 4 months before admission to the ETU was 459/μL on April 24, 2014. His only measured CD4 cell count after recovery from EVD was 529/μL on August 9, 2016. He reported compliance with his ART regimen and denied any serious illness since the time of his HIV diagnosis.

**Figure:**
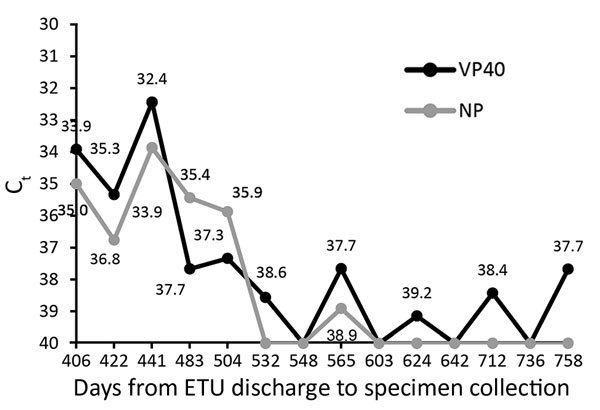
Ebola virus RNA detected by RT-PCR in semen samples from an HIV-positive survivor (48-year-old man) of Ebola virus disease, Monrovia, Liberia, 2009–2016. RT-PCR cycle threshold (C_t_) values for Ebola virus VP40 and NP gene targets are reported by days from the patient’s discharge from the ETU to collection of a semen specimen. A gene target is considered detected if the C_t_ is <40. If gene amplification is not demonstrated within 40 cycles, then the gene target is considered undetectable and no C_t_ is reported. All undetectable results are indicated as C_t_ values of 40. CD4 cell counts per microliter were 46 on October 20, 2009; 48 on November 12, 2009; 358 on March 25, 2010; 358 on April 22, 2010; 563 on November 22, 2010; 824 on January 22, 2013; 459 on April 24, 2014; and 529 on August 9, 2016. The patient had a CD4 cell count of 529/μL 699 days after discharge from the Ebola treatment unit. On November 12, 2009, the patient was given an ART regimen of zidovudine/lamivudine/nevirapine. On April 22, 2010, the ART regimen changed to zidovudine/lamivudine/lopinavir plus ritonavir. ART, antiretroviral therapy; C_t_, cycle threshold; ETU, Ebola treatment unit; NP, nucleoprotein, RT-PCR, reverse transcription PCR; VP40; viral structural protein 40.

The patient enrolled in the Men’s Health Screening Program on October 21, 2015. Per program protocol, his semen was tested every 4 weeks for Ebola virus by RT-PCR using described methods (*2*). Specimens are considered positive if viral structural protein 40 gene and nucleoprotein gene targets of Ebola virus have C_t_ values <40, and indeterminate if only 1 gene target has a C_t_ <40. Since his enrollment in the program, the semen of the patient has been positive for Ebola virus RNA up to 565 days after he was discharged from the ETU. C_t_ values plateaued to indeterminate for samples up to 758 days after discharge (Figure). Although detection of Ebola virus RNA by RT-PCR does not necessarily indicate the presence of infectious virus, a previous study reported Ebola virus infectivity by RT-PCR–positive human semen samples in immunodeficient mice (*3*).

The prolonged period during which Ebola virus RNA was detected in this patient adds to evidence (*2**–**4*) that there is heterogeneity in duration of Ebola virus persistence in semen among survivors of EVD. Although etiology of this heterogeneity is unclear, possible explanations for this patient include age-associated effects (*2*), attenuated clearance caused by dual HIV infection, immunosuppression from etiologies other than HIV, severity of acute illness, or unknown host genetic factors. Although the patient had an adequate CD4 cell count, chronic inflammation, immune system dysregulation, and accelerated immunoscenescence in well-controlled HIV patients have been described and are clinically manifested as early cardiovascular disease, neurocognitive disorders, metabolic syndrome, and non–AIDS-associated cancers (*5*). Therefore, co-infection with HIV might play a role in persistence of Ebola virus in semen, despite an adequate clinical response to ART.

Because HIV infection is treatable and testing is readily available in West Africa, semen testing programs for Ebola virus should consider offering HIV testing to male survivors of EVD with persistently detectable Ebola virus in semen. Furthermore, HIV care was interrupted during the Ebola outbreak in West Africa because of closure of clinics and interruption of ART distribution (*6*). This case- patient had a favorable outcome for EVD despite being HIV positive, which emphasizes the need for continuing treatment for HIV infection in the setting of a large-scale Ebola outbreak. In addition, this case highlights the need for a better understanding of the role that co-infection with HIV might play in persistent detection of Ebola virus RNA in male survivors of EVD.

## References

[1] Mate SE, Kugelman JR, Nyenswah TG, Ladner JT, Wiley MR, Cordier-Lassalle T, et al. Molecular evidence of sexual transmission of Ebola virus. N Engl J Med. 2015;373:2448–54. 10.1056/NEJMoa1509773 26465384 PMC4711355

[2] Soka MJ, Choi MJ, Baller A, White S, Rogers E, Purpura LJ, et al. Prevention of sexual transmission of Ebola in Liberia through a national semen testing and counselling programme for survivors: an analysis of Ebola virus RNA results and behavioural data. Lancet Glob Health. 2016;4:e736–43. 10.1016/S2214-109X(16)30175-9 27596037

[3] Sissoko D, Duraffour S, Kerber R, Kolie JS, Beavogui AH, Camara AM, et al. Persistence and clearance of Ebola virus RNA from seminal fluid of Ebola virus disease survivors: a longitudinal analysis and modelling study. Lancet Glob Health. 2017;5:e80–8. 10.1016/S2214-109X(16)30243-1 27955791

[4] Diallo B, Sissoko D, Loman NJ, Bah HA, Bah H, Worrell MC, et al. Resurgence of Ebola virus disease in Guinea linked to a survivor with virus persistence in seminal fluid for more than 500 days. Clin Infect Dis. 2016;63:1353–6. 10.1093/cid/ciw601 27585800 PMC5091350

[5] Deeks SG. HIV infection, inflammation, immunosenescence, and aging. Annu Rev Med. 2011;62:141–55. 10.1146/annurev-med-042909-093756 21090961 PMC3759035

[6] Hira S, Piot P. The counter effects of the Ebola epidemic on control and treatment of HIV/AIDS, tuberculosis, and malaria in West Africa. AIDS. 2016;30:2555–9. 10.1097/QAD.0000000000001231 27525552

